# Comparison of Intraoperative Results of Simple Open and Laparoscopic Nephrectomies in the Treatment of Benign Renal Pathologies in a First-Level Center in Mexico City

**DOI:** 10.7759/cureus.68142

**Published:** 2024-08-29

**Authors:** Alejandro Martinez-Esteban, Natalia M Barron-Cervantes, Kevin J Fuentes-Calvo, Sara F Arechavala-Lopez, Roxana Ramos-Carpinteyro, J Jesus Cendejas-Gomez, Carlos E Méndez-Probst

**Affiliations:** 1 General and Gastrointestinal Surgery Service, Fundacion Clinica Medica Sur, Mexico City, MEX; 2 Urology, Instituto Nacional de Ciencias Médicas y Nutrición Salvador Zubirán, Mexico City, MEX

**Keywords:** intraoperative results, hospital stay, hemorrhage, benign renal pathologies, laparoscopic nephrectomy, simple open nephrectomy

## Abstract

Objectives: To assess the efficacy and safety of simple open versus laparoscopic nephrectomies for treating benign renal pathologies, with a focus on comparing the prevalence of surgical complications at a first-level center in Mexico City.

Methods: A retrospective analysis spanning 2010-2020 was conducted where all patients undergoing simple nephrectomy for benign conditions were included and stratified into open and laparoscopic surgery groups. Variables analyzed included urological history, laboratory findings, surgical outcomes, complications, and histopathological results. Statistical comparisons employed Student's t-test for means and the chi-square test for frequencies. Additionally, binary logistic regression was utilized to identify predictors associated with conversion from laparoscopic to open surgery.

Results: The laparoscopic approach showed significant advantages in intraoperative bleeding (p=0.008) and intensive care unit stay (p=0.04). The conversion rate from laparoscopic to open surgery was 19.23%, with no significant risk factors identified for conversion.

Conclusions: Laparoscopic simple nephrectomy proves to be a secure and effective method in specialized urological centers with skilled surgeons, offering superior intraoperative outcomes compared to open surgery. It effectively reduces intraoperative hemorrhage, minimizes blood transfusion needs, and shortens hospital stays. Nonetheless, challenges such as equipment availability, costs, and surgeon expertise must be addressed. Further research focused on postoperative complications is crucial to advocate for broader adoption of laparoscopic nephrectomy as the preferred standard for treating relevant urological conditions, emphasizing substantial advantages over traditional open approaches.

## Introduction

For more than two decades, the preferred surgical approach for simple nephrectomies has been the open approach, but the laparoscopic way has been a point of controversy. It has been shown in some studies that laparoscopic surgery can have intraoperative surgical advantages, such as less bleeding and quick recovery. By definition, a simple nephrectomy (SN) involves the removal of a kidney by extracting anything within the Gerota fascia, leaving everything outside of this structure, such as the adrenal gland and the regional lymph nodes intact. Although the name may incorrectly suggest that this surgical procedure is a simple intervention, it is known that this surgery is highly complex and can be associated with both intra- and post-operative complications [1]. In order to perform this surgical procedure, two approaches may be used: open surgery and laparoscopic surgery. Throughout the years, many research papers have been published describing the incidence and prevalence of complications associated with each technique.

Since its implementation, multiple studies comparing its efficiency, effectiveness, and associated complications have been published worldwide. However, a comparative study of this type has never been carried out in a first-level center in Mexico City. With only two studies published in Mexico, it was found that the national data did not agree with the international literature. This incongruity prompted the initiation of a retrospective analysis aimed at scrutinizing the prevalence of intraoperative complications associated with SN, contrasting the open and laparoscopic approaches. This investigation was conducted within one of the nation's foremost primary referral centers, Instituto Nacional de Ciencias Médicas y Nutrición Salvador Zubirán, in order to provide localized insights into this pertinent clinical domain.

Hypothesis

Null hypothesis: There is a difference between the prevalence of the variables studied when comparing the group with the open approach against the group with the surgical laparoscopic approach.

Alternative hypothesis: There is no difference between the prevalence of the variables studied when comparing the group with the open approach against the group with the surgical laparoscopic approach.

Objectives

Primary objective: To compare the efficacy and safety of simple open and laparoscopic nephrectomies in the treatment of benign renal pathologies comparing the intra- and post-surgical complications’ prevalence in both groups in a first-level center in Mexico City.

Secondary objectives: To define the frequency of presentation of each variable studied in each group: laparoscopic and open and to define if the prevalence presented in the literature will be the same as the one presented in a first-level center in Mexico City.

## Materials and methods

This is a single-center retrospective study of male and female patients who underwent simple nephrectomy for benign pathology between 2010 and 2020 in the Urology Department of Instituto Nacional de Ciencias Médicas y Nutrición Salvador Zubirán. The patients were divided into two groups depending on the surgical approach they underwent: open and laparoscopic. The analysis covers intra- and post-surgical variables of the patients, such as urological history, laboratory studies, surgical findings, complications, and histopathological results of the specimen. The variables studied include intraoperative bleeding evaluated in milliliters (mL) and days of stay in the intensive care unit in both groups. Statistical tests included mean comparisons using Student's t-test and frequency comparisons using the chi-square test. Additionally, a binary logistic regression was performed to identify factors associated with conversion from laparoscopic to open surgery.

Inclusion and exclusion criteria

Inclusion criteria: All male and female patients who signed the consent form and underwent simple open and laparoscopic nephrectomy for benign pathology between 2010 and 2020 in the Urology Department of Instituto Nacional de Ciencias Médicas y Nutrición Salvador Zubirán.

Exclusion criteria: All male and female patients that underwent simple open and laparoscopic nephrectomy for malign pathology. All cases that were before or after the time frame studied. All patients underwent open or laparoscopic radical nephrectomy.

Sampling method

The sampling method utilized in this study was the probability sampling method. The subtype selected was simple random sampling, this method was chosen because the sample size was large, and the item was chosen randomly, which allowed it to be a representative sampling of the population studied.

## Results

**Figure 1:**
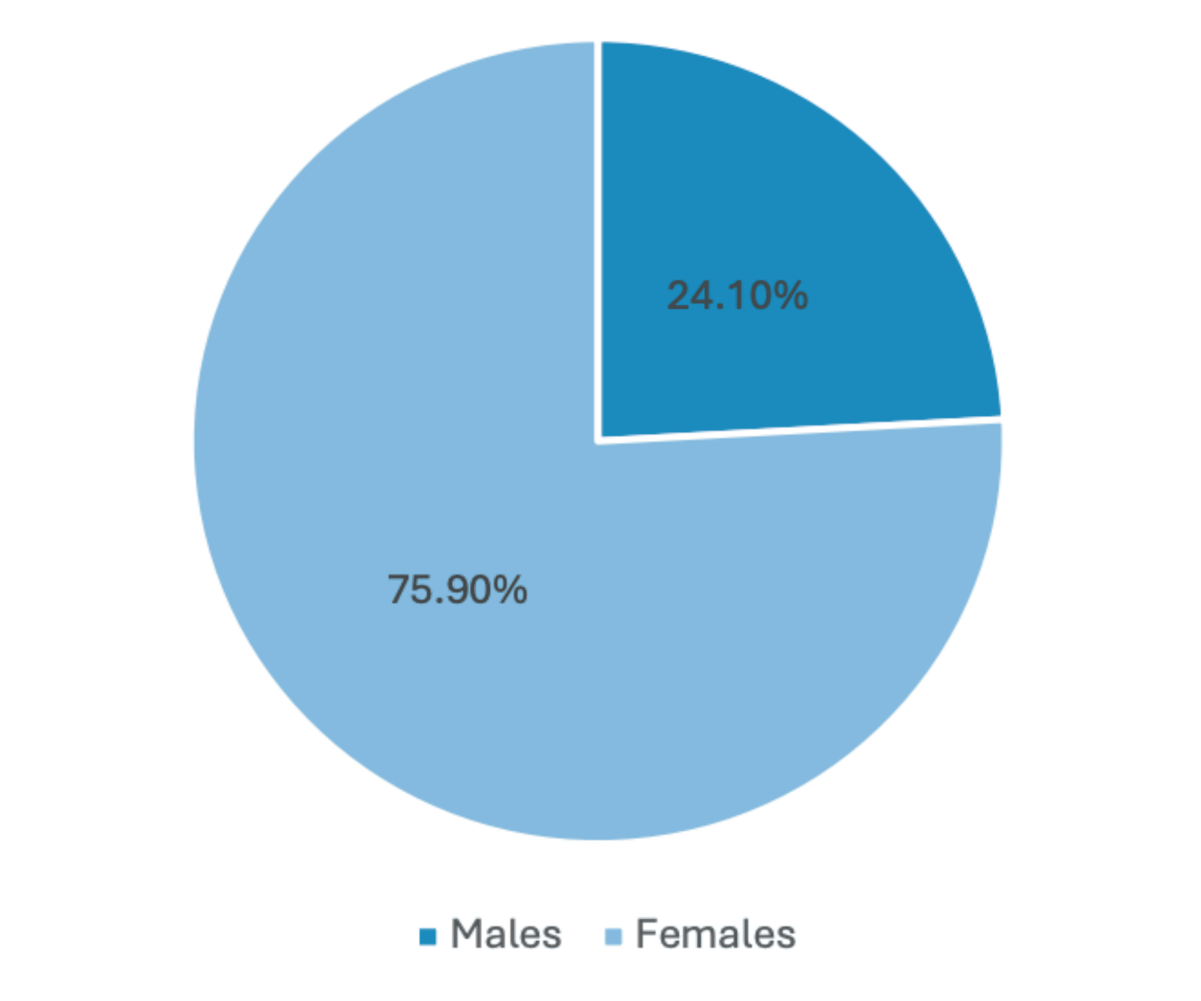
Population distribution based on sex. A graphical representation in the form of a pie chart depicting a total of 87 patients was studied: 21 men (24.1%) and 66 women (75.9%).

**Figure 2:**
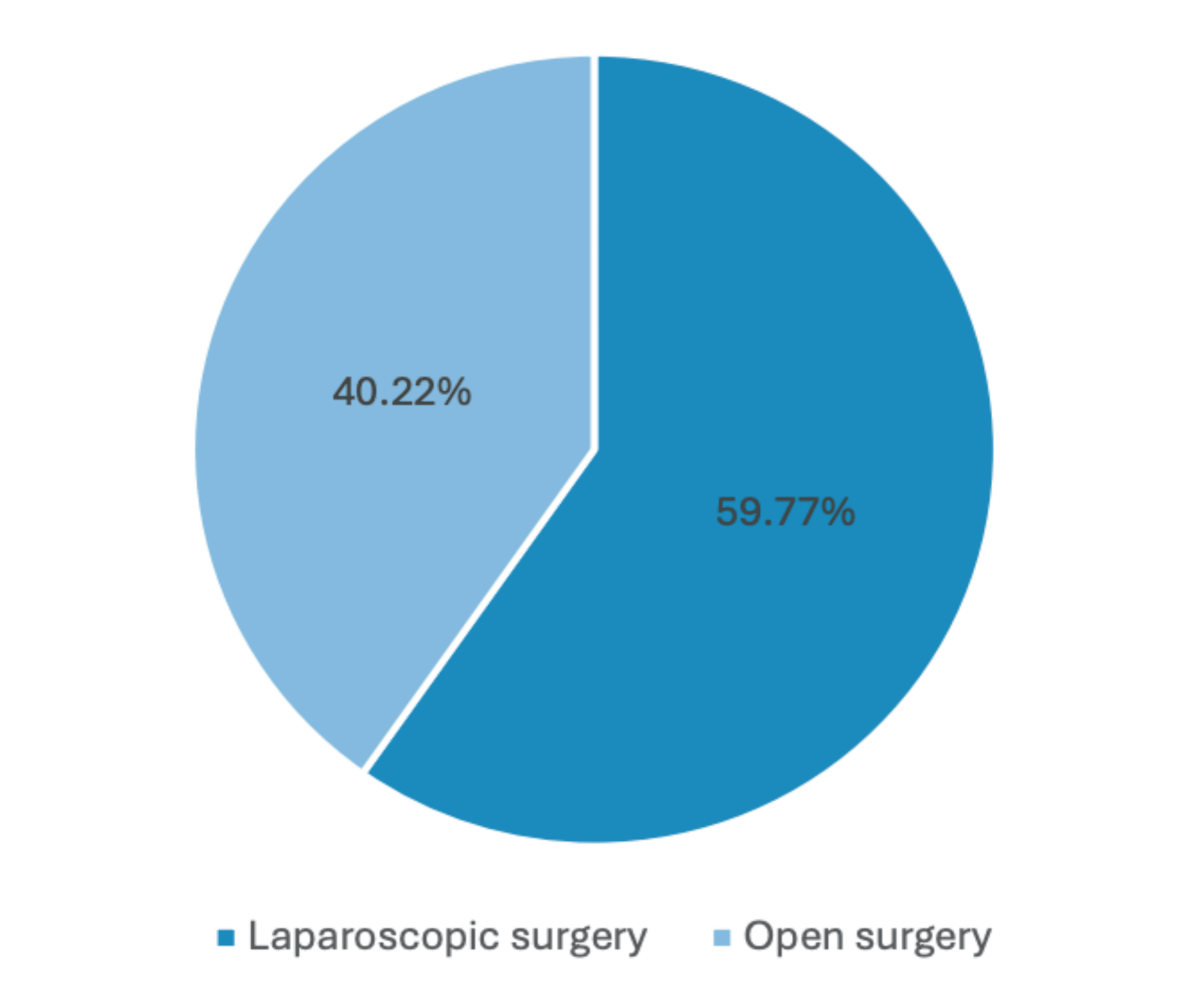
Distribution of the types of surgery. A graphical representation in the form of a pie chart depicting the distribution of types of surgical procedures, where 40.22% of all the cases studied were approached by open surgery and 59.77% by laparoscopic surgery.

A total of 87 patients were studied, 21 men (24.1%) and 66 women (75.9%) (Figure 1). Of these, 35 patients (40.22%) were approached by open surgery, and 52 patients (59.77%) were treated by a laparoscopic approach (Figure 2). Of all the patients managed through a laparoscopic approach, 11 (12.64%) were men, and 41 were women (47.12%). The open surgery approach was utilized in 10 men (11.49%) and 25 women (28.73%). Conversion from a laparoscopic to an open approach was needed in 17 patients because of bad visibility associated with increased visceral fat and previous surgical incisions that conditioned surgical adhesions. Basic clinico-demographic details are exposed (Table 1).

**Table 1:** Basic clinico-demographic details. Table presenting the basic clinico-demographic details of the population being studied. Columns are divided in three main groups, the patients who underwent a laparoscopic approach, patients who underwent an open surgery approach, and the total of patients who present the clinico-demographic detail presented. Each clinico-demographic detail is presented in a different row. Subheads inside the table include: sex, surgical conversion from laparoscopic to open surgery approach, and complications studied. For sex and surgical conversion, the number of patients (n) that presented that characteristic are shown, and next to them the percentage of presentation is shown. For the complications studied the mean, the interquartile range (IQR) and the p value are included.

Basic clinico-demographic details	Laparoscopic approach		Open surgery approach		Total of patients
	n	percentage (%)	n	percentage (%)	n
All participants	52	59.77	35	40.22	87
Sex					
Male	11	12.64	10	11.49	21
Female	41	47.12	25	28.73	66
Surgical conversion from laparoscopic to open surgery approach					
All converted cases	17	19.54	0	0	17
Complications studied	Mean	IQR	Mean	IQR	p value
Intraoperative bleeding (ml)	97.45	77.12-118.25	114.56	88.34-127.22	0.008
Stay in the Intensive Care Unit (days)	7.12	7.52-6.11	8.09	8.27-7.99	0.04

**Figure 3:**
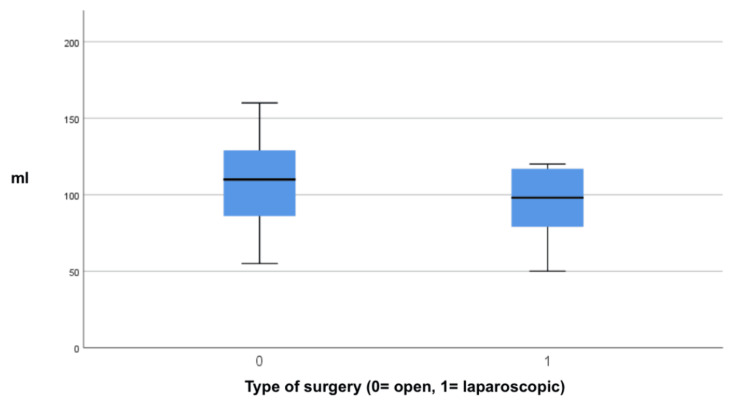
Forrest plot of intraoperative bleeding. A graphical representation in the form of a box plot depicting a mean intraoperative bleeding of 97.45 mL (IQR: 77.12-118.25) was recorded in the group undergoing laparoscopic surgery (1), in contrast to a mean of 114.56 mL (IQR: 88.34-127.22) in the group undergoing open surgery (0). This difference was statistically significant (p=0.008, 95% CI).

The benefits of the laparoscopic approach over open surgery were found in less intraoperative bleeding (p=0.008) and shorter need to stay in the ICU (p=0.04). The mean intraoperative bleeding of 97.45 mL (IQR: 77.12-118.25) was recorded in the group undergoing laparoscopic surgery, in contrast to a mean of 114.56 mL (IQR: 88.34-127.22) in the group undergoing open surgery. This difference was statistically significant (p=0.008, 95% confidence interval (CI)) (Figure 3).

**Figure 4:**
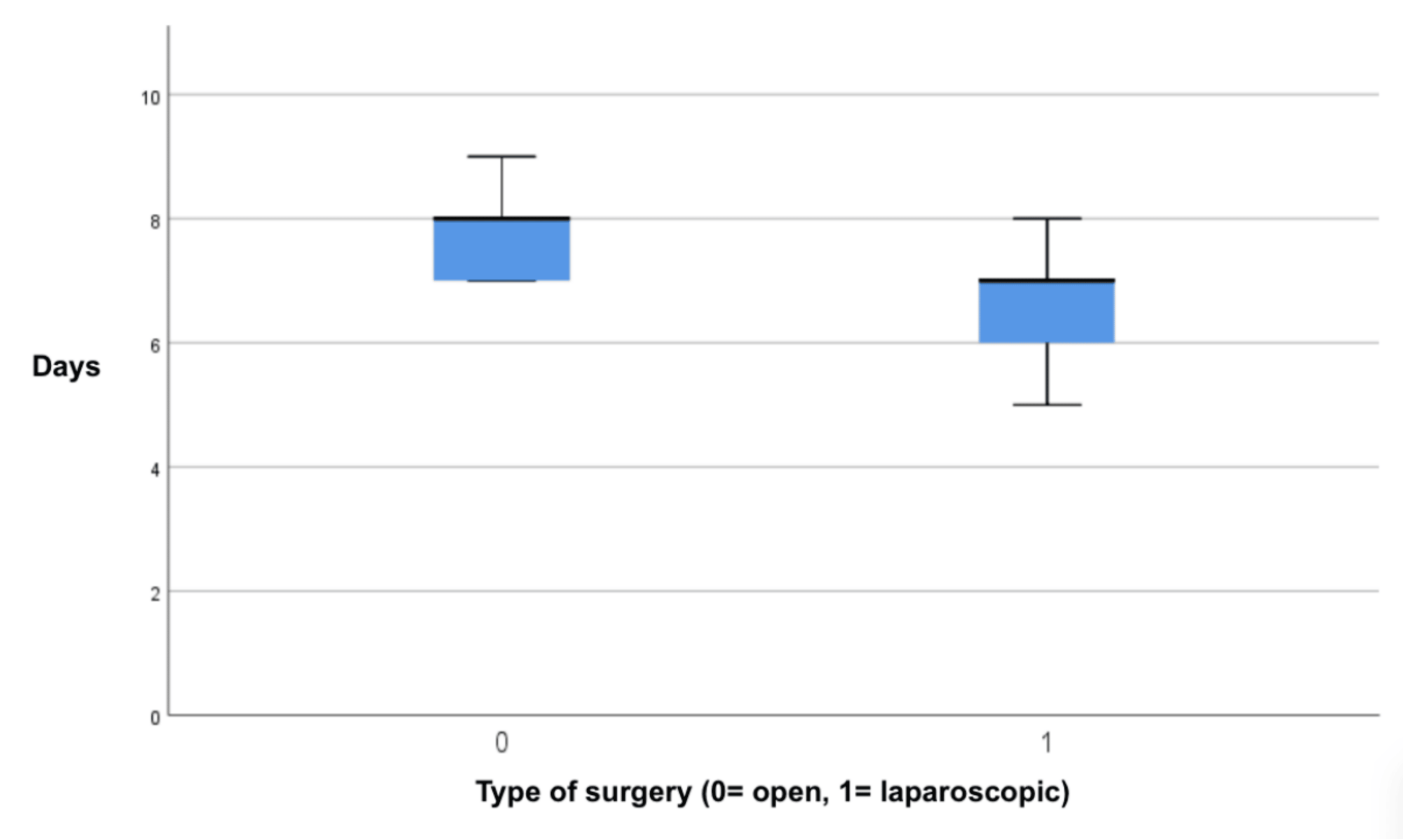
Forrest plot of the length of stay in the intensive care unit. A graphical representation in the form of a box plot depicting a reduction in the number of days of intensive care unit stay in the group that underwent laparoscopic surgery (1) compared to the group that underwent open surgery (0). In the laparoscopic group, the average length of stay was 7.12 days (IQR: 7.52-6.11), while in the open surgery group, it was 8.09 days (IQR: 8.27-7.99). This difference was statistically significant (p=0.04, 95% CI).

There was a reduction in the number of days of the ICU stay in the group that underwent laparoscopic surgery compared to the group that underwent open surgery. In the laparoscopic group, the average length of stay was 7.12 days (IQR: 7.52-6.11), while in the open surgery group, it was 8.09 days (IQR: 8.27-7.99). This difference was statistically significant (p=0.04, 95% CI) (Figure 4).

## Discussion

SN is the gold standard treatment for benign renal pathology. During this surgical procedure, the kidney is removed, leaving every structure outside the Gerota fascia intact, which includes the ipsilateral adrenal gland and the regional lymph nodes [1]. For many decades, the only available option was to perform this intervention by performing an open subcostal incision on the affected side to access the retroperitoneum and Gerota's fascia, allowing the surgeon to eventually remove the dysfunctional kidney. However, with the discovery of laparoscopic surgery, many other specialties sought to use less invasive procedures to perform conventional surgeries. It is important to acknowledge that over the passage of time, advancements in laparoscopic surgical techniques have facilitated notable enhancements, thereby enabling the undertaking of increasingly intricate surgical procedures, such as SN [2]. The first time a laparoscopic nephrectomy was performed for the treatment of a benign renal disease was in 1991 by Dr. Clayman M.D. [3], at that time this intervention was reported to decrease morbidity, length of hospital stay, and pain and improve in general the post-operative recovery [4]. As mentioned earlier, this procedure kept improving in a way it evolved from multiple port nephrectomies to a single-port nephrectomy, also known as laparoendoscopic single-site surgery (LESS), which was first performed in 2007 by Dr. Raman M.D. [5].

A meta-analysis performed by the Mayo Clinic in 2004 described that laparoscopic surgeries presented advantages over open surgery, such as decreased postoperative pain, shortened hospital stay, more rapid return to normal activities, and improved cosmetics [6]. In the results, it was proven that laparoscopic surgery presented a mortality rate of 0%, and there was only one case of intraoperative complication, which was the presentation of an iatrogenic injury in the inferior epigastric artery. The most common intraoperative complications were vascular, which required the conversion of laparoscopic to open nephrectomies in some cases. Additionally, the only postoperative complications presented were urinary retention and port-side hernia [6]. It is important to mention that there exist two possible approaches for a laparoscopic SN, which include a retroperitoneal and a transperitoneal approach [4]. Nowadays, the indications for an SN include chronic pyelonephritis, obstructive or reflux nephropathy, renal tuberculosis, multicystic dysplastic kidney, renovascular hypertension, acquired renal cystic disease in dialysis patients, nephrosclerosis, symptomatic patients with autosomal dominant polycystic kidney disease, and post kidney transplantation hypertension [6]. Relative contraindications involve intra-abdominal adhesions that may pose a technical difficulty during insufflation, trocar placement, and dissection. In these cases, a retroperitoneoscopic approach is preferred [7].

There are two studies previously carried out in first and third-level centers in Mexico. These studies were carried out at the Centro Médico Nacional Siglo XXI Specialty Hospital [8] in Mexico City, one of the largest first-level centers in the country. The other study, carried out in a tertiary center, was carried out at the Unidad Médica de Alta Especialidad (UMAE) No. 25 [9] in Nuevo León. The significance of these studies lies in their examination of diverse populations across various regions within the country. Both studies independently reached a consistent conclusion that there exists no discernible variance in the incidence and prevalence of complications associated with SN, irrespective of whether the surgical approach employed was open or laparoscopic [8,9]. An important point to highlight is that in this paper, as well as in the literature from the country, the length of hospital stays is generally prolonged. This extension is directly associated with the fact that, being public hospitals, discharges are often delayed due to bureaucratic issues rather than medical reasons. This situation tends to be quite common throughout the country. In general, findings from this statistical analysis stand in contrast to the prevailing international literature on the subject. The results presented in this study contrast with the conclusions presented by the previously mentioned studies, which allows us to accept that there is a difference at the national level between third-, second-, and first-level centers. Our data showed that there is a statistically significant difference with p values ​​less than 0.05 in intraoperative complications, such as intraoperative bleeding and length of stay in the intensive care unit, both being lower in the group with the laparoscopic approach.

Study limitations

Firstly, the research focuses exclusively on two variables: days of intensive care unit (ICU) stay and intraoperative bleeding. This narrow scope means that the findings are restricted to these variables and may not be generalizable to other related factors. Additionally, the study is limited by its examination of a select population from a reference hospital, which consists of complex patients with multiple comorbidities. This specific patient group may introduce bias, potentially skewing the results and influencing both primary outcomes, the length of ICU stay, and intraoperative bleeding. At the same time, it is important to mention that there is a larger female population in this particular hospital, which causes more cases to occur in female patients than in male patients. New studies must be carried out to confirm the correlation applies to other complications associated with these types of surgeries. Similarly, studies should be carried out that include a homogeneous population that is more similar to the general population of the country and not the population that is treated in a reference hospital.

## Conclusions

Based on the findings of this study, it is deduced that laparoscopic simple nephrectomy represents a secure and viable approach within urological referral centers that possess proficient surgeons adept in minimally invasive procedures. Notably, this technique yields superior intraoperative outcomes and diminishes postoperative complications, particularly reducing the incidence of intraoperative hemorrhage, minimizing the necessity for blood transfusions, and shortening hospitalization durations, when contrasted with conventional open surgery. Nevertheless, it is essential to acknowledge that challenges such as equipment availability, associated costs, and surgeon proficiency remain significant drawbacks. Thus, further investigation into postoperative complications is warranted to advocate for the widespread adoption of laparoscopic simple nephrectomy as the preferred standard of care for relevant urological conditions. Because of the aforementioned differences, given the findings delineated in this study, it can be inferred that there exists a substantial disparity between employing the laparoscopic approach and the open approach.
